# The extracellular N-terminal domain of G-protein coupled receptor 83 regulates signaling properties and is an intramolecular inverse agonist

**DOI:** 10.1186/1756-0500-7-913

**Published:** 2014-12-16

**Authors:** Anne Müller, Brinja Leinweber, Jana Fischer, Timo D Müller, Annette Grüters, Matthias H Tschöp, Vera Knäuper, Heike Biebermann, Gunnar Kleinau

**Affiliations:** Institute of Experimental Pediatric Endocrinology, Charité-Universitätsmedizin Berlin, Augustenburger Platz 1, Ostring 3, 13353 Berlin, Germany; Institute for Diabetes and Obesity, Helmholtz Center Munich, Munich, Germany, and Division of Metabolic Diseases, Department of Medicine, Technische Universität München, Munich, Germany; School of Dentistry College of Biomedical & Life Sciences, Cardiff University, Wales, UK

**Keywords:** G-protein coupled receptor 83, Signaling mechanism, Inverse agonist, Antagonist, Constitutive activation

## Abstract

**Background:**

Recently, the orphan G-protein coupled receptor 83 (GPR83) was identified as a new participant in body weight regulation. This receptor is highly expressed in the hypothalamic arcuate nucleus and is regulated in response to nutrient availability. *Gpr83* knock-out mice are protected from diet-induced obesity. Moreover, in a previous study, we designed and characterized several artificial constitutively activating mutations (CAMs) in GPR83. A particular CAM was located in the extracellular N-terminal domain (eNDo) that is highly conserved among GPR83 orthologs. This suggests the contribution of this receptor part into regulation of signaling, which needed a more detailed investigation.

**Findings:**

In this present study, therefore, we further explored the role of the eNDo in regulating GPR83-signaling and demonstrate a proof-of-principle approach in that deletion mutants are characterized by a strong increase in basal Gq/11-mediated signaling, whilst none of the additionally characterized signaling pathways (Gs, Gi, G12/13) were activated by the N-terminal deletion variants. Of note, we detected basal GPR83 MAPK-activity of the wild type receptor, which was not increased in the deletion variants.

**Conclusions:**

Finally, the extracellular portion of GPR83 has a strong regulatory function on this receptor. A suppressive - inverse agonistic - effect of the eNDo on GPR83 signaling activity is demonstrated here, which also suggests a putative link between extracellular receptor activation and proteolytic cleavage. These new insights highlight important aspects of GPR83-regulation and might open options in the development of tools to modulate GPR83-signaling.

## Introduction

G-protein coupled receptors (GPCRs) are involved in regulating the flow of information across membranes, they are tuning components of the cellular-physiological machinery and serve as hubs for signal transduction between different biological units [1]. This information flow subsequently results in specific physiological or, in the event of dysregulation, pathophysiological reactions [2]. GPCRs and their respective ligands can be multi-key players, which are often simultaneously related to different processes and might be responsible to synchronize or coordinate several processes (e.g. metabolism, reproduction and neurobiology).

Many different GPCRs have been identified to be involved in the regulation of metabolism and body weight such as the melanocortin-4 receptor (MC4R) or the ghrelin receptor (GHSR) (reviews [3, 4]). Of note, obesity is the most common preceding health condition leading to many concomitant health disorders, including type 2 diabetes mellitus, hypertension, arteriosclerosis, several types of cancer, polycystic ovarian syndrome or sleep apnea [5]. Obesity should be considered as a disease [6], which has globally attained epidemic proportions over the last decades and is no longer restricted to developed countries [4].

The G-protein coupled receptor 83 (GPR83) [7, 8] was recently identified as a new determinant involved in body weight regulation [9]. This orphan receptor is most abundantly expressed in the thymus and brain [8, 10, 11]. GPR83 has been previously found to be involved in the control of circulating adiponectin levels [7]. The endogenous ligand of GPR83 remains to be identified.

Our recently published studies have deciphered several new functional and physiological properties of GPR83. Briefly, GPR83 is involved in systemic energy metabolism via ghrelin-dependent and ghrelin-independent mechanisms [9]. This receptor is highly expressed in the hypothalamic arcuate nucleus, and it has been demonstrated that hypothalamic expression of Gpr83 is dependent on nutrient availability. Moreover, Gpr83 expression is decreased in obese compared to lean mice and constitutes homodimers but also has the capacity for heterodimerization (e g. with the ghrelin receptor) [12]. Basal Gq/11 related signaling activity has been shown for Gpr83 as well as slight activation or stabilization of the active conformation using Zn(II) supplementation. We recently designed several artificial constitutively activating mutations (CAMs) [12], whereby a particular CAM was located in the extracellular N-terminal domain (eNDo) that is highly conserved among GPR83 orthologs. In this current study, we further explored the contribution of the eNDo region on GPR83-signaling in order to advance insights into the GPR83-signaling mechanism. For this purpose, deletion constructs of the eNDo were designed and functionally characterized.

## Findings

**Figure 1:**
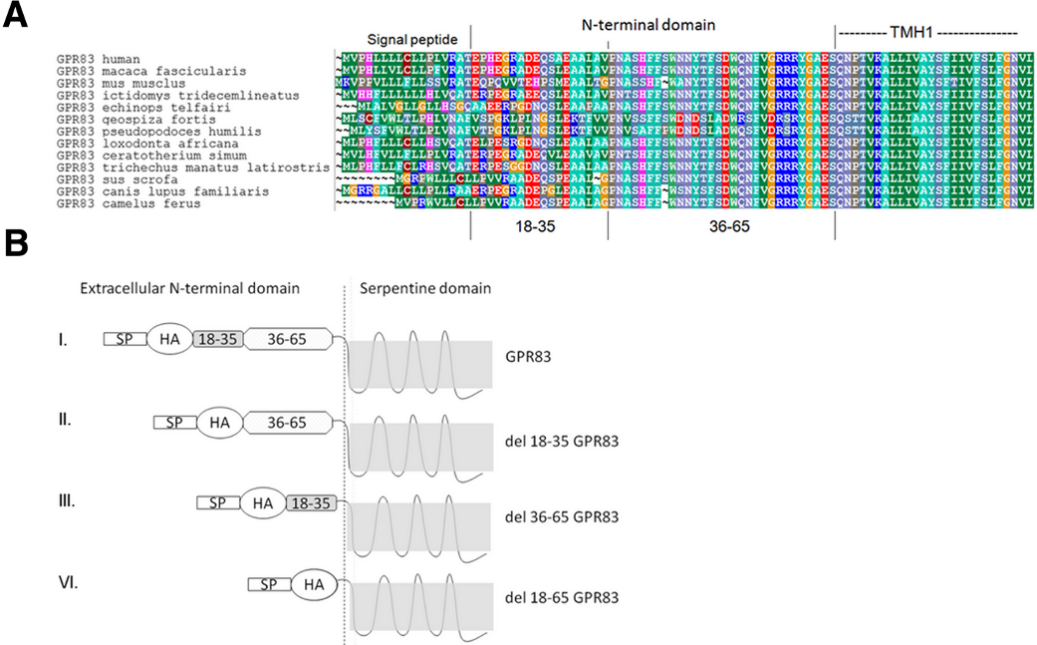
**Sequence comparison of GPR83 othologs and designed GPR83 variants A) Alignment of N-terminal amino acids of GPR83 orthologs for comparison and identification of sequence conservation.** Regions of conservation can be recognized by the overlapping colors. High conservation is also indicative for a specific fold and/or function. It is evident that especially the second half of the N-terminal tail (between positions 36–65) is highly conserved among the compared variants. Different colors of amino acids indicate their biophysical properties: green – hydrophobic, blue – positively charged, red – negatively charged. The alignment was visualized using *BioEdit*. **B)** Schematic representation of GPR83 deletion mutants: The experimentally deleted parts are highlighted: a. deletion 18–35, b. deletion 36–65, c. deletion 18–65. The position of the signal peptide is indicated as SP and the hemagglutinin tag with HA.

In this study, we aimed to unravel the role of the GPR83 N-terminal domain on receptor function, which was initially tested using deletion constructs. All constructs retained the GPR83 signal peptide and had an adjacent HA-tag to monitor cell surface expression levels. We deleted residues 18–35 or the entire eNDo (residues 18–65) as well as an internal peptide sequence consisting of residues 36–65 (Figure 1A and B). These three GPR83 constructs were functionally characterized with regard to their basal signaling activity and cell surface expression levels. In addition, a previously identified Gq/11-CAM (single point mutation H331A [12] in the transmembrane helix (TMH) 7) was used for comparison.

### The cell surface expression level of the Gpr83 deletion constructs is differently modified

**Figure 2:**
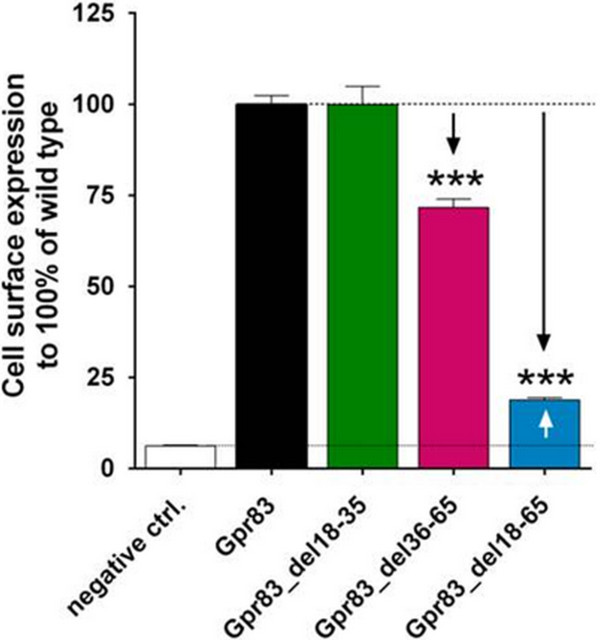
**Cell surface expression levels of different N-terminal GPR83 deletion mutants compared to wild type GPR83 which was set to 100% (absorption (492/620): 0.31 ± 0.01).** The *Gpr83* variants were detected using an HA-ELISA system as previously reported [12]. Untagged GPR83 served as the negative control. Data were assessed from a minimum of three independent experiments, each performed at least in triplicate, and are represented as mean + SEM. ***p ≤ 0.001 (unpaired t-test, two-tailed).

**Figure 3:**
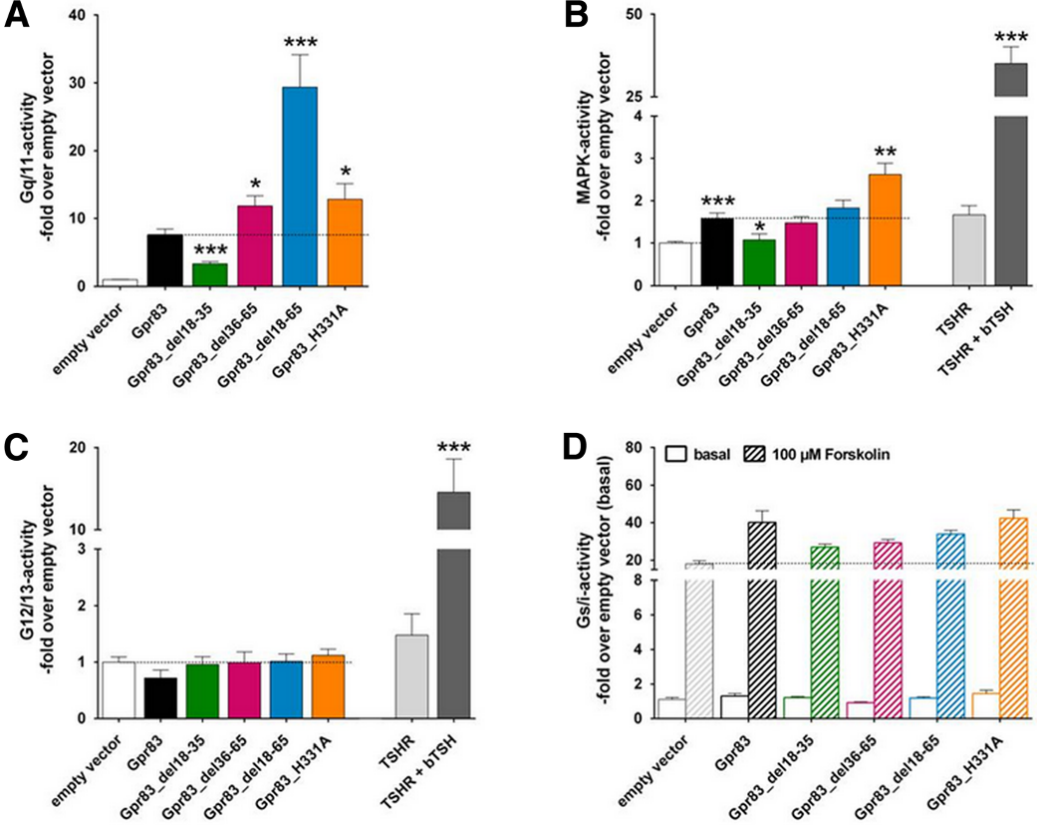
**Different N-terminal GPR83 deletion mutants were functionally characterized and compared with the wild type GPR83 (A/B) or an empty vector control (pcDps, B/C/D).** Shown are Gq/11-activation in **A)**, MAPK-activation in **B)**, G12/13-activation in **C)** and Gs/Gi-signaling properties in **D)**. Wild type GPR83 serves as a positive control for Gq/11-signaling **(A)**. The TSH-stimulated TSHR serves as a positive control for MAPK- and G12/13-signaling **(B/C)**. Forskolin-stimulation shows Gs-activity of the used cell line by activating adenylyl cyclase. A decreased value of forskolin-stimulation in comparison to the empty vector control would indicate inhibitory Gi-activity. Data were evaluated from a minimum of three independent experiments, each performed at least in triplicate. Data were calculated as fold over the empty vector control, set to 1 **(A**: 4828.67 ± 1165.71, **B**: 1334800 ± 326986.69, **C**: 2095559.89 ± 447919.48 relative light units; **D**: 2.03 ± 0.15 nM cAMP). Data represent mean + SEM. *p ≤ 0.05, **p ≤ 0.01, ***p ≤ 0.001 (unpaired t-test, two-tailed).

Cell surface expression of the deletion constructs (Figure 2) were either found to be comparable with wild type GPR83 expression level (deletion variant del18-35) or slightly decreased (del36-65, 72% of wild type receptor). On the other hand, the cell surface expression level for del18-65 was observed to be strongly decreased to 19% of wild type level, which is most likely related to the modified structural features and a high level of induced constitutive signaling activity (Figure 3).

### Deletion of the entire extracellular domain leads to high constitutive Gq/11-activation

Despite the detection of low levels of receptor cell-surface expression, removal of the entire eNDo of GPR83 resulted in an approximately four-fold increase in basal Gq/11-signaling activity (Figure 3A), indicating a dramatic increase in basal activity. This increase in basal activity was also confirmed for a receptor variant without an N-terminally fused tag (not shown), which excludes an influence of the receptor-function by this modification.

Deletion of the C-terminal part of the eNDo (between positions 36–65, Figure 3A) constitutively activates GPR83 to levels comparable to the H331A-CAM in TMH7, with a 1.4-fold increase over basal activity (Figure 3A). In contrast, deletion of the first half of the eNDo (del18-35) decreased basal Gq/11-signaling to approximately 60% of wild type GPR83 (Figure 3A), which indicates an inverse agonistic effect of this deletion on basal GPR83-activity.

### GPR83 basal MAPK-signaling can be increased by a single point mutation in TMH7

We next investigated whether GPR83 was able to activate MAPK-signaling. Indeed, GPR83 was found to mediate a basal level of MAPK-activity (Figure 3B). Surprisingly, deletion of position 18–35 resulted in impaired basal MAPK-signaling (Figure 3B), which was also observed for Gq/11-signaling. In contrast, the removal of the entire eNDo or the internal peptide (position 36–65) did not alter MAPK/ERK-activation in comparison to wild type GPR83 (Figure 3B). A two-fold increase in MAPK-activity was detected for the H331A CAM, indicating that MAPK-signaling may be regulated by residues in the transmembrane region. Thus, the structural requirements for activation of MAPK and Gq/11 signaling by GPR83 are distinct and regulated by different domains.

In addition, we analyzed G12/13- as well as Gs/Gi-signaling but could not detect any activity for wild type GPR83 or for any of the N-terminal deletion mutants investigated (Figures 3C/D). In order to detect Gi-activity, the adenylyl cyclase enhancer forskolin was used to produce increased cAMP levels. Any mutation causing constitutive Gi activity of a receptor variant would decrease forskolin-stimulated cAMP values below the empty vector control, this was however not observed, thus Gi coupling can be excluded.

## Discussion

### The entire N-terminal part of GPR83 is likely a domain that functions as an inverse agonist of Gq/11-mediated signaling

**Figure 4:**
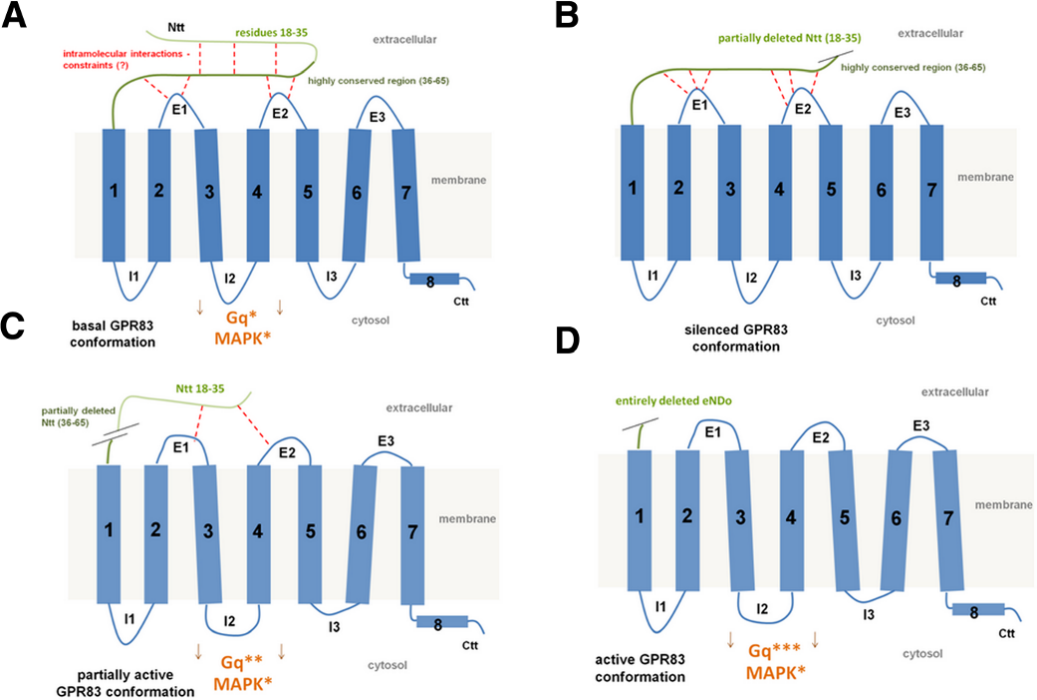
**Structural scheme of GPR83 and variants with indicated findings and derived hypotheses. A)** In wild type GPR83, the N-terminal domain (eNDo) is stabilized intramolecularly by side-chain interactions between specific amino acids (red broken lines). The eNDo might have a defined structural fold, which is indicated by high amino acid conservation throughout GPR83 orthologs (Figure 1). The eNDo most likely interacts with the extracellular loops (E’s 1–3) and these interactions probably maintain the basal state. **B)** In contrast, deletion of residues 18–35, a partial deletion, increases the inverse agonistic effect of the remaining eNDo residues on GPR83 and suppresses basal activity, by presumptively stabilizing the inactive conformation. **C)** Deletion of residues 35–65 of the eNDo leads to slight receptor activation, possibly due to the partial loss of intramolecular interactions with the extracellular loops. **D)** The GPR83 becomes highly active following removal of the entire N-terminal domain. Ctt = C-terminal tail, I = intracellular loop, E = extracellular loop.

The complete deletion of the N-terminal extracellular part of GPR83 specifically induces the constitutive activation of Gq/11-signaling by this receptor. In contrast, Gs, Gi or G12/13-related pathways or MAPK-signaling were not activated. This indicates that the extracellular N-terminus of GPR83 has evolved to stabilize an inactive receptor conformation and thus serves as an intramolecular inverse agonist (schematized in Figure 4A). Of note, the GPR83 variant del18-35 revealed cell surface expression levels similar to the wild type receptor, while the highly active del18-65 full deletion variant is characterized by low cell surface expression levels of approximately 20% of the wild type receptor (Figure 2). The high conservation of amino acids throughout several GPR83 orthologs, especially in the second N-terminal half of GPR83 (Figure 1A), supports the assumption of a functionally important region that might also be characterized by a specific structural fold. We assume that interactions from the N-terminal domain to additional regions of the receptor exist, most likely with the extracellular loops in order to maintain a (partially) inactive basal receptor conformation (Figure 4). Removal of the entire eNDo would lead to an active receptor conformation as N-terminal interactions with the accessible extracellular receptor loops should be released (Figure 4). Interestingly, activation of TSHR by deletion of the entire eNDo or extracellular fragments leads also to constitutive signaling activity [13–15], and suggests an inverse agonistic function of the TSHR-eNDo.

### Different N-terminal receptor fragments with specific properties

At this stage, we cannot provide data to explain the decreased basal signaling activity caused by the deletion of residues 18–35, an effect which contradicts the findings for the deletion of residues 36–65 (Figure 4B). It can only be speculated that the deletion of residues 18–35 strengthens the interactions between residues 36–65 e.g. with the extracellular loops of GPR83, resulting in an enforced inactive conformation leading to a further loss of basal activity.

Interestingly, only the removal of the entire eNDo was found to result in a high activation, whereas the other two shorter deletion mutants analyzed demonstrated either only weak activity or a lack of constitutive activity (Figure 4B, C). The data point to a more complex interplay between the different receptor parts either intra- or intermolecularly.

### The MAPK pathway and Gq/11 signaling are regulated differently

For the first time, we demonstrate basal MAPK activity for GPR83, which is impaired upon deletion of residues 18–35. In contrast to the findings for Gq/11-signaling activity, removal of the entire eNDo or residues 35–65 displayed constitutive MAPK-activity levels seen for wild type GPR83. However, the H331A-CAM mutation, located in TMH7, showed constitutive activation of the MAPK pathway. In conclusion, activation of Gq/11 by GPR83 can be induced by modification of the extracellular domain, but MAPK-signaling is not induced by this event and can be forced by a single CAM in the transmembrane region. The data may indicate differences in the detailed activation mechanism of diverse signaling pathways activated by GPR83.

### Conclusions and open questions

Finally, the extracellular portion of GPR83 has a strong regulatory function on this receptor. A suppressive - inverse agonistic - effect of the eNDo on GPR83 signaling activity is demonstrated here, which also suggests a putative link between extracellular receptor activation and proteolytic cleavage *in vivo*.

As the work presented here has opened several new perspectives on this receptor with regards to signaling mechanisms and regulation, the following summary highlights open questions of great interest in warranting their further research:

-  We do not know the exact interplay between the extracellular parts of GPR83 or relationship between single amino acids and activity regulation. It would be of great interest to further narrow down the particularly important structural determinants of the inverse agonist activity observed for the eNDo domain.
-  The maximal level of GPR83-signaling activity remains to be identified, due to an unknown agonistic endogenous ligand or activation mechanism. The constitutive activity attained following full-length deletion of the eNDo may or may not be maximal, and we cannot exclude that other pathways as found here may be activated by the physiological ligand.
-  Potentially, the eNDo domain may also act like a ligand recognition motif, as has been observed for the ligands TSH and thyrostimulin of the TSHR (reviewed in [16]), which is also a class A GPCR with a long extracellular N-terminal region. However, we cannot address this in the absence of an endogenous ligand for GPR83.
-  The contribution of a dimeric constellation (intermolecular interactions) on signaling regulation remains unknown. Our findings may also conceivably be related to a modified interplay between protomeric GPR83 interactions in dimeric (oligomeric) constellations.
-  For endogenous GPR83-activation, we hypothesize that a specific N-terminal region of GPR83 might be recognized and cleaved/clipped by a protease. Potential enzyme candidates are disintegrin, metalloproteinase ADAM 10 (A Disintegrin And Metalloproteinase 10) [17] or trypsin [18], whereby ADAM 10 does potentially cleave but not activate TSHR (reviewed in [19]). Moreover, positively charged amino acid side chains are recognition motifs for trypsin [20]. Such positively charged residues (such as arginines) are also present in a cluster-like manner in the eNDo of GPR83 (Figure 1).
-  In addition, it is known that the G-protein coupled protease-activated receptors (PARs) become active following cleavage by serine proteases such as thrombin [21]. In conclusion, tests with proteolytic enzymes are required in order to unravel their potential contribution on GPR83 activation or signaling.
-  For both the TSHR [13] and PARs, it has been described that fragments of the eNDo (with (PARs) or without (TSHR) cleavage) function as an intramolecular agonist. This contribution of the GPR83-eNDo for the endogenous activation mechanism remains ambiguous.
-  Other GPCRs coupled to Gq/11 (e.g. the angiotensin II receptor or the α-adrenergic 2 receptor [22, 23]) have exhibited their involvement in stimulating the activation of metalloproteinases for shedding of further proteins [22] and/or activating signaling pathways such as MAPK [23]. Therefore, a future interest would be to investigate the contribution of ADAMs as downstream effectors or activating proteases of GPR83.

Finally, our findings open new perspectives for GPR83 related research by advanced insights into structure-function relationships.

## Materials and methods

### Construction of wild type and mutant receptors

Cloning of *Gpr83* was performed as recently described [12]. *Gpr83* was amplified from murine hypothalamic cDNA and, as control for functional assays, thyroid stimulating hormone receptor (TSHR) () was cloned from human thyroid cDNA. Receptor-cDNAs were cloned into the pcDps expression vector. A hemagglutinin tag was cloned downstream of the signal peptide of Gpr83 (SP-HA, see Figure 1B for schematic representation). The H331A mutation of Gpr83 was generated by site-directed mutagenesis. The deletion mutants were created by successive fractional cloning (Figure 1B). Automatic sequencing was used to determine the accuracy of the PCR-derived products. The pGL4.30[luc2P/NFAT-RE/Hygro], pGL4.33[luc2P/SRE/Hygro] and pGL4.34[luc2P/SRF-RE/Hygro] reporter constructs, co-transfected for Gq/11, MAPK (mitogen-activated protein kinase) or G12/13 (Rho A) determination, were purchased from Promega (Mannheim, Germany).

### Cell culture and transfection

COS-7 cells were cultured in Dulbecco’s modified medium (DMEM/Biochrom, Berlin, Germany), whereas HEK293 cells were cultured in minimal Essential medium (MEM/Biochrom, Berlin, Germany), and both were supplemented with 10% fetal bovine serum, 100 U/ml penicillin, and 100 μg/ml streptomycin and incubated at 37°C in a humidified 7% CO2 incubator. For measurement of Gq/11-, MAPK- and G12/13-activity via reporter gene assay, HEK293 cells were seeded into 96-well plates (1.5 × 10^4^ cells/well), coated with poly-L-lysin (Biochrom, Berlin, Germany). For Gs/Gi determination, COS-7 cells were seeded into 96-well plates (0.9 × 10^4^ cells/well). Transfection was performed with 41.7 ng of receptor plasmid-DNA/well and 0.5 μL Metafectene™/well (Biontex, Martinsried, Germany) one day after seeding. For Gq/11, MAPK and G12/13 determination equal amounts of the appropriate reporter construct, containing the firefly luciferase gene, was co-transfected.

### Cell surface expression studies

Cell surface expression studies of wild type GPR83 and designed constructs were carried out in COS-7 cells and were performed using an ELISA system that detects HA-tagged receptors. A tag-less GPR83 served as a negative control (detailed description in [12]). Seventy two hours after transfection cells were washed two times with Dulbecco’s phosphate buffered saline (DPBS, Biochrom, Berlin) and fixed for 30 min. in 4% formaldehyde in DPBS followed by two times washing in DPBS. After incubation in blocking buffer (10% FCS supplemented DMEM) for 1 h at 37°C followed by a washing step in DPBS cells were incubated for 2 h in blocking buffer with 1 μg/ml biotin labelled anti-HA monoclonal antibody (Roche, Mannheim) at 37°C followed by three washes in DPBS and incubation in blocking buffer with 1 μg/ml streptavidin labelled peroxidase (Dianova, Hamburg) at 37°C for 1 h followed by three times washing. The color reaction was carried out with 0.1% H_2_O_2_ and 10 μg o-phenylendiamine in 0.1 M citric acid and 0.1 M Na_2_HPO_4_ at pH 5.2. The reaction was stopped after 10 min. with 1 M Na_2_SO_3_ in 1 M HCl. Colorimetry was carried out using an anthos reader 2001 (anthos labtech instruments, Salzburg).

### Measurement of Gq/11, MAPK and G12/13 via reporter gene assay

Activity of Gq/11-, MAPK- and G12/13-signaling was determined 48 hours after transfection. The TSHR stimulated with 100 mU/mL bovine thyroid stimulating hormone (TSH, purchased from Sigma-Aldrich, Taufkirchen, Germany) served as a positive control in each case [24, 25]. Following six hours of stimulation, cells were lysed with 50 μl/well of 1x Passive Lysis Buffer (Promega). Pathway activities were determined by luciferase activity according to the manufacturer’s instructions (Promega).

### Determination of Gs- or Gi-signaling

Intracellular cAMP levels to determine Gs- or Gi-activation were measured in COS-7 cells in the presence of 1 mM 3-isobutyl-1-methylxanthine (Sigma-Aldrich, Munich, Germany) using AlphaScreen technology [26]. The hTSHR stimulated with 100 mU/mL bovine TSH (Sigma-Aldrich) served as Gs positive control [24, 25]. To investigate Gi-activity, cells were treated with 50 μM forskolin (Sigma-Aldrich). After stimulation with forskolin for 45 minutes, cell lysis (50 μL/well lysis buffer) and cAMP measurement were performed as previously described [12].

### Data analysis

Statistical analyses were performed using the statistical tools implemented in Graph Pad Prism, version 5 (GraphPad Software, San Diego, California, USA).

## Abbreviations

GPCR: G-protein coupled receptor
GPR83: G-protein coupled receptor 83
TSHR: Thyrotropin receptor
ICL: Intracellular loop
ECL: Extracellular loop
TMH: Transmembrane helix
Ntt: N-terminal tail
eNDo: Extracellular N-terminal domain
MC4R: Melanocortin-4 receptor
GHSR: Ghrelin receptor
CAM: Constitutively activating mutation
PAR: Protease-activated receptor.
